# Selection by parasitoid females among closely related hosts based on volatiles: Identifying relevant chemical cues

**DOI:** 10.1002/ece3.3877

**Published:** 2018-02-19

**Authors:** Lisa Fors, Raimondas Mozuraitis, Laima Blažytė‐Čereškienė, Thomas A. Verschut, Peter A. Hambäck

**Affiliations:** ^1^ Department of Ecology Environment and Plant Sciences Stockholm University Stockholm Sweden; ^2^ Laboratory of Chemical and Behavioural Ecology Institute of Ecology Nature Research Centre Vilnius Lithuania; ^3^ Present address: Department of Zoology Stockholm University Stockholm Sweden

**Keywords:** *Asecodes parviclava*, electrophysiology, headspace volatile collection, host–parasitoid system, olfactometer

## Abstract

Parasitoid fitness is influenced by the ability to overcome host defense strategies and by the ability of parasitoid females to select high‐quality host individuals. When females are unable to differentiate among hosts, their fitness will decrease with an increasing abundance of resistant hosts. To understand the effect of mixed host populations on female fitness, it is therefore necessary to investigate the ability of female parasitoids to select among hosts. Here, we used behavioral assays, headspace volatile collection, and electrophysiology to study the ability of *Asecodes parviclava* to use olfactory cues to select between a susceptible host (*Galerucella calmariensis*) and a resistant host (*Galerucella pusilla*) from a distance. Our studies show that parasitoid females have the capacity to distinguish the two hosts and that the selection behavior is acquired through experiences during earlier life stages. Further, we identified two volatiles (α‐terpinolene and [*E*]‐β‐ocimene) which amounts differ between the two plant–herbivore systems and that caused behavioral and electrophysiological responses. The consequence of this selection behavior is that females have the capacity to avoid laying eggs in *G. pusilla*, where the egg mortality is higher due to much stronger immune responses toward *A. parviclava* than in larvae of *G*. *calmariensis*.

## 1 INTRODUCTION

Evolution of parasitoids in parasitoid–host interactions depends not only on their ability to overcome host defense strategies but also on the ability of parasitoid females to select the highest quality individuals of either the same or different host species (van Alphen & Drijver, 1982; Desneux, Blahnik, Delebecque, & Heimpel, 2012; Dupas, Dubuffet, Carton, & Poirie, 2009; Rolff, 2002; Rolff & Kraaijeveld, 2001). If parasitoid females are able to differentiate between hosts with weak and strong immune responses, they will gain from selecting the host with the lowest resistance. Because this selection behavior reduces egg laying in the resistant host, and thus increases parasitoid survival, we expect relatively weaker selection for higher virulence in parasitoid populations (Dupas et al., 2009; Hood, Egan, & Feder, 2012). On the other hand, if female parasitoids are unable to differentiate between resistant and susceptible hosts, their egg survival would decrease with the relative abundance of resistant hosts in the area. In this case, we expect relatively stronger selection on parasitoids to evolve increased virulence to overcome the immune response of the resistant host (Kraaijeveld & Godfray, 2009). Thus, to understand the strength of selection on higher virulence in parasitoids, it is important to study the ability of females to select among hosts of different resistance level. Preference–performance experiments with parasitoid females generally indicate that female preference is related to offspring performance (Dubuffet, Alvarez, Drezen, Van Alphen, & Poirie, 2006; Dupas et al., 2009), but in some cases, parasitoid females have been found to select hosts where offspring have a very low survival (Heimpel, Neuhauser, & Hoogendoorn, 2003). Because these studies generally do not identify the sensory cues involved, it is unknown if the inability of parasitoid females to select among hosts may be due to a lack of suitable cues. Moreover, it is also less known whether females that do select among hosts based on offspring performance require direct contact with the host or can also detect host quality differences from a distance.

The most common sources of sensory information used by parasitoids for host selection are olfactory and gustatory cues (Vinson, 1976). There is a variety of chemical cues deriving from the host (Fatouros, Dicke, Mumm, Meiners, & Hilker, 2008) or from the host's habitat that can be used by parasitoids to find and select among hosts (Vet & Dicke, 1992). The most reliable cues for finding herbivore hosts are often induced volatiles that change qualitatively or quantitatively when herbivores feed on plant tissue (Dicke et al., 1990; Du, Poppy, & Powell, 1996; Du et al., 1998; Godfray, 1994; Takabayashi et al., 1998). Various studies have shown that parasitoid females use volatile profiles to differentiate between host and nonhost species (e.g., de Boer, Hordijk, Posthumus, & Dicke, 2008; de Rijk, Dicke, & Poelman, 2013), but most cases involve nonhost species that belong to a different feeding guild than the host species. There are also cases where the host and nonhost are closely related. For instance, De Moraes, Lewis, Pare, Alborn, & Tumlinson, 1998 have shown that the parasitic wasp *Cardiochiles nigriceps* is able to differentiate between the volatile profiles from the host *Heliothis virescens* and the congeneric nonhost *H. zea* when feeding on the same tobacco plants.

In this study, we investigated the role of volatiles in the host selection of the parasitoid *Asecodes parviclava* (Hymenoptera: Eulophidae) between larvae of the closely related hosts *Galerucella calmariensis* and *Galerucella pusilla* (Coleoptera: Chrysomelidae), feeding on the same host plant (purple loosestrife, *Lythrum salicaria*). The two hosts have very different effects on parasitoid survival, where *G. calmariensis* shows a very weak immune response and *G. pusilla* a very strong immune response when attacked by *A. parviclava* (Fors, Markus, Theopold, Ericson, & Hambäck, 2016; Fors, Markus, Theopold, & Hambäck, 2014). As a result, attacks on *G. calmariensis* are usually successful and lead to high offspring survival, whereas attacks on *G. pusilla* mostly lead to encapsulation of parasitoid eggs and low offspring survival. Due to these different effects on parasitoid survival, we would expect parasitoid females to develop a capacity to differentiate between the two hosts, but the capacity to do so may be limited as the two hosts are monophagous on the same plant species and may cause releases of similar volatile profiles.

We used a combination of behavioral assays, electrophysiological responses, and headspace volatile collection, to study the role of volatiles in the host selection of *A. parviclava*. Previous parasitism studies indicate that females respond very fast to the presence of host larvae in the vicinity, even when larvae are concealed (Fors, unpublished data), suggesting that olfactory cues are involved in host finding. We therefore focused the first part of our study on the ability of *A. parviclava* females to select between the two larval species (*G. calmariensis* and *G. pusilla*), when feeding on the host plant (*L. salicaria*), using a Y‐tube olfactometer. Due to the low survival of parasitoids in *G. pusilla*, we were unable to get a sufficient number of parasitoids from this species, restricting our ability to study the role of natal experience for this host–parasitoid pair. To approach this question, we instead used parasitoids originating from *Galerucella tenella,* a third host species of *A. parviclava*, in addition to the parasitoids originating from *G. calmariensis*. Parasitoids from these two hosts were collected from the same localities, and previous studies suggest that these parasitoids are one interbreeding population (Hambäck et al., 2013). As *G. tenella* does not feed on *L. salicaria,* parasitoids originating from *G. tenella* have no natal experience from either study species or from their host plant. Therefore, differences in the response of parasitoids originating from *G. calmariensis* and *G. tenella* would indicate that the parasitoids may be using information acquired during previous life stages when selecting hosts (van Emden, Sponagl, Baker, Ganguly, & Douloumpaka, 1996; Verschut, Blažytė‐Čereškienė, Apšegaitė, Mozūraitis, & Hambäck, 2017), creating a chemical legacy effect (Giunti et al., 2015; Vet & Groenewold, 1990).

In the second part of the study, we examined likely candidate compounds that could explain the observed selection behavior by parasitoid females in the Y‐tube olfactometer. For this purpose, we first identified the five compounds that provide the strongest signal‐to‐noise ratio when comparing the volatile profiles from the two larval species feeding on the plant. In the next step, we tested the ability of parasitoid females originating from *G. calmariensis* to identify these five compounds in a Y‐tube experiment. In the final step, we used electroantennographic detection as a measure of the ability of parasitoid antennae to detect these compounds and compared the responses to a range of other compounds. These experiments confirm the result from the behavioral studies, suggesting that *A. parviclava* females are able to differentiate between the two host species based on volatiles. We also identify two compounds that may explain the host selection behavior.

## 2 MATERIALS AND METHODS

### 2.1 Study species

**Figure 1:**
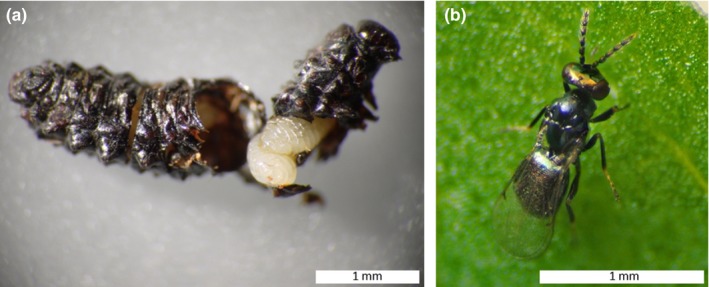
(a) Mummified larva of *Galerucella calmariensis* (Coleoptera: Chrysomelidae), showing pupae of the parasitic wasp *Asecodes parviclava* (Hymenoptera: Eulophidae) inside. (b) Adult female of *A. parviclava*. Scale bars: 1 mm

Two species of *Galerucella* leaf beetles (Coleoptera: Chrysomelidae), *G. calmariensis* (L.) and *G. pusilla* (Duftschmid), are the focal species in this study. Both species use *L. salicaria* (Lythraceae) exclusively as host plant for feeding and oviposition (Hambäck, 2004)*. L*. *salicaria* (purple loosestrife) is a perennial herb, native to large parts of Eurasia, growing in moist and coastal areas. In Sweden, *L*. *salicaria* occurs throughout the country in the south and in the coastal area further north. Both beetle species occur from the south up until Sundsvall [N 62º, E17º], whereas *G*. *calmariensis* is also common further north. Mating takes place in early summer, and the eggs are deposited on the host plant, hatching after a few weeks. Both larvae and adults feed on all aboveground parts of the plant, which can sometimes lead to severe damage. The larvae pupate after 2–4 weeks, and the new adults emerge from the pupae 2–3 weeks later (Hambäck, 2004). The third beetle species in this study, *G. tenella*, has a similar life cycle as the other species but uses another main host plant species (*Filipendula ulmaria*). In the south, *G. calmariensis* and *G. pusilla* are often found at the same sites, and often even on the same plant individual, while *G. tenella* and its host plant are often spatially separated from the other two *Galerucella* species. In the north, where *G. pusilla* is lacking, *G. tenella* and *G. calmariensis* often occur close together. Larvae of all three *Galerucella* species are attacked by the parasitic wasp *A. parviclava* Thompson (Hymenoptera: Eulophidae) (Hambäck et al., 2013; Hansson & Hambäck, 2013). The three *Galerucella* species are the only known hosts for *A*. *parviclava*. The parasitoid attacks the host in the larval stage, laying one or more eggs inside the larva. The parasitoid larvae consume their host from within, making it unable to form a normal pupa at the time for pupation. Instead, parasitized larvae turn into mummified black shells in which the parasitoid larvae pupate and from which the adults subsequently emerge (Figure 1).

For the experiments of this study, we collected adult beetles of *G. pusilla* and *G. calmariensis* in the field in mid‐May and held them in the laboratory for mating and oviposition. We used two sites in the southeast of Sweden [N59º90, E18º20] for collection of both species. The eggs were kept in the laboratory until hatching, and the larvae used for the behavioral assays were all in the second instar and of approximately the same size. The parasitoids were collected when hatching from previous season's mummified larvae of *G. calmariensis* and *G. tenella*, which had been overwintered outdoors in individual vials. The vials were brought indoors 3–4 weeks before the expected use (as this is the usual time for the parasitoids to wake up), kept under controlled, stable temperatures (20°C during day [15 hr] and 17°C during night [9 hr]), and checked twice each day for emerging individuals. Upon emergence, the parasitoids were transferred to 250‐ml plastic containers with a feeding station (sugar solution) and a droplet of water. Only mated females were used in the experiments, within 7 days from emergence. All parasitoids originated from two northern sites of *G. calmariensis* and *G. tenella* [N63º50, E20º40], as parasitoid abundance in the south was low. Hence, the parasitoids originate in different localities than the larvae, thus avoiding the possibility of local adaptation. The plant material used for the experimental treatments was collected from potted plants (at least 3 months of age) that were maintained in an outdoor enclosure. Prior to the tests, all plants were checked thoroughly for any herbivorous insects (which were removed) and then kept in the greenhouse until the time for the experiments.

### 2.2 Behavioral assays

**Figure 2:**
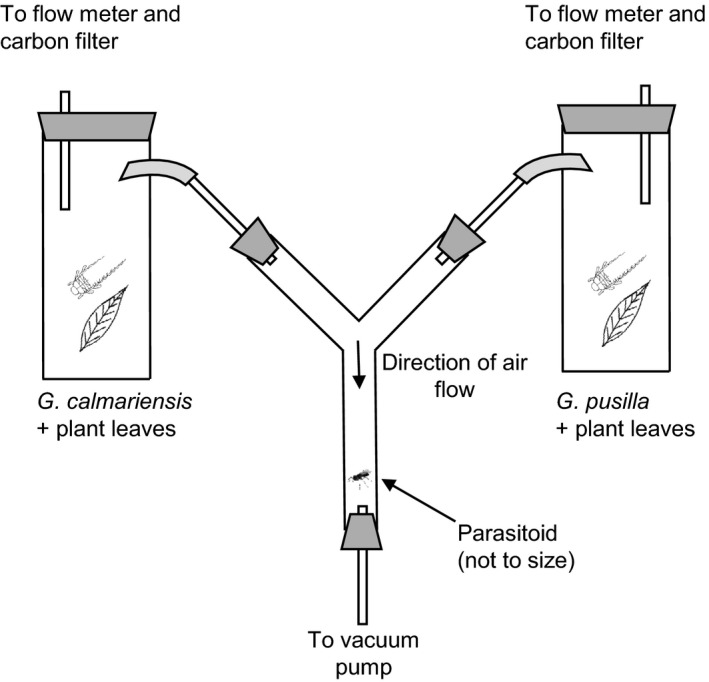
General setup of the Y‐tube glass olfactometers used to test the behavioral responses in *Asecodes parviclava* to different volatiles

Behavioral responses of *A. parviclava* were studied in Y‐tube glass olfactometers (Humi Glas AB, Lund, Sweden). We performed two sets of trials to study the behavioral responses by mated *A. parviclava* females to volatiles from the two larvae–plant combinations. In the first set, parasitoids originating from *G. calmariensis* and *G. tenella* were allowed to select between larvae of *G calmariensis* and *G. pusilla* when feeding on the host plant *L*. *salicaria*. We included parasitoids originating from *G. tenella* in these trials to investigate the chemical legacy effect, as these parasitoids had not been exposed to volatiles from either *G. calmariensis* or *G. pusilla* during the larval or pupal stages. For each test, branches of 10–15 cm (approximately the same total leaf area) were cut from *L*. *salicaria,* and four larvae of either species were placed on each branch. The branches with larvae were placed in separate gas‐washing bottles (Figure 2), and the larvae were allowed to feed for 30 min prior to the experiment to ensure acclimatization and the release of sufficient odors. In the second set, female parasitoids from *G. calmariensis* were allowed to respond to synthetic chemical compounds (or solvent as a control). Chemical compounds were selected based on differences in the headspace volatiles from the two larval species when feeding on the host plant (see results for details), and concentrations were selected based on observed concentrations in the GC‐MS analysis. Single volatile compounds or mixtures of compounds were dissolved in paraffin oil (CAS No 8012‐95‐1, Sigma‐Aldrich) and released from a 2‐cm^2^ filter paper (grade 3, Munktell Filter AB, Falun, Sweden) in a gas‐washing bottle, against a volatile background of mechanically damaged leaves. The volatile background consisted of five detached leaves of approximately the same total leaf area that were damaged with an entomological pin before placing them in the bottles. The volatile background was included to simulate a natural situation, as females may respond less to specific compounds when these compounds are released in solitude. Filter paper impregnated with 10 μl pure paraffin oil was used as a control. Volatiles were not used longer than 1 hr, as longer use would cause reduced emittance.

The stem of the Y‐tube used in the assays measured 100 mm, with an inner diameter of 18 mm, and an angle of 90° between the two arms which measured 75 mm. The parasitoids were introduced to the olfactometer through a 4 mm hole at the beginning of the stem. The end of the stem was connected to an air inlet secured with a glass ground joint to a diaphragm pump (MZ 2C, Vacuubrand GmbH, Wertheim, Germany), in order to draw air mixed with test odors through the entire system. The different treatments were placed in the gas‐washing bottles (⌀ 4.5 cm × 15 cm height, 250 ml), connected by Teflon tubing (⌀ 5 mm) to an air inlet secured in a glass ground joint at both arms of the Y‐tube. The inlet of the gas‐washing bottle was connected to a flow meter (Kytola Instruments Model E, Muurame, Finland) to stabilize the airflow at approximately 30 ml/s for each arm of the Y‐tube. All air entering the system was purified with an activated carbon filter placed at the inlet of the flow meter. The experiments were performed in a constant temperature (21 ± 2°C), darkened room with the Y‐tubes lit from a height of 50 cm by LED‐lights covered with opaque acryl glass to ensure a diffuse distribution of the light roughly corresponding to natural light. At the beginning of each trial, the parasitoid was introduced to the Y‐tube and given 2 min to acclimatize without any airflow. Once the parasitoids had acclimatized, the introduction hole was closed to ensure a steady airflow through the system. Each parasitoid was observed for a maximum of 10 min or until it made a decisive choice. The choice was recorded by a human observer when the parasitoid had passed two‐thirds of an arm of the Y‐tube and stayed there for at least 5 s. Measurements of parasitoids that did not make a decisive choice after 10 min were discarded, following the protocol in Stenberg, Heijari, Holopainen, & Ericson, 2007. We used each Y‐tube for six consecutive tests and then thoroughly cleaned them with a mild odorless detergent and 70% ethanol, letting them dry in an oven at 200°C to eliminate any odors from the glass surface. In order to avoid positional effects, we rotated the Y‐tubes after 2–3 observations and the gas‐washing bottles after each washing.

### 2.3 Identification of volatiles

To compare the volatiles released from the two plant–herbivore systems, headspace volatile samples were collected from potted *L. salicaria* plants (at least 3 months of age) infested with third instar larvae (10 per replicate) of either *G. calmariensis* or *G. pusilla*. A branch with 5–7 leaves (approximately the same leaf biomass) was used for each setup, and a new plant was prepared for each individual volatile collection. The plant part used (together with larvae) was enclosed in a polyester cooking bag (Toppits, 25 × 40 cm), whereas the rest of the potted plant was kept intact outside the bag. In addition, blank sample volatile collections were used to distinguish between the volatiles released by the plant–herbivore system and those released by empty bags. Volatiles were collected for 24 hr by solid phase microextraction (SPME) (Pawliszyn, 1997), using fibers coated with 65 μm polydimethylsiloxane–divinylbenzene (Supelco, Sigma‐Aldrich group, PA, USA). The volatiles were collected under controlled, stable temperatures (22°C during day [18 hr] and 19°C during night [6 hr], RH = 60%), using the same headspace volume and with an addition of internal standard to monitor sampling capacity of the fibers. The SPME fibers were purified at 225°C for 2 min in a gas chromatograph (GC) injector before each odor sampling. After routine purification of the fiber, the needle of the syringe was used to pierce the wall of the polyester bag, and the purified fiber was exposed to a headspace. After collection, the fiber was retracted into the needle, removed from the bag, and transferred to a GC injector for 0.5 min to desorb volatiles. Under these circumstances, SPME is an excellent and very sensitive technique. However, it is not possible to compare quantities of different compounds within the same sample without the use of labeled standards, due to different affinities of compounds on the fiber and different vapor pressures of the target compounds (Romeo, 2009).

The analysis of the collected volatiles was performed using a Varian 3400 gas chromatograph (GC) (Varian, Palo Alto, CA, USA) connected to a Finnigan SSQ 7000 mass spectrometer (MS) (Thermo‐Finnigan, San Jose, CA, USA). A DB‐wax silica capillary column with an internal diameter of 0.25 mm and a film thickness of 0.25 μm (J & W Scientific, Folsom, CA, USA) was used. The temperature program started at 40°C (1 min), initially increasing 5°C/min up to 200°C, then by 10°C/min up to 230°C, and thereafter held isothermally at 230°C for 15 min. The split/splitless injector temperature was 225°C with a splitless period of 30 sec. The carrier gas was helium, with a flow rate of 0.9 ml/min. Electron ionization mass spectra were determined at 70 eV with the ion source at 150°C in the range 30–400 Daltons. The chromatographic data of volatiles collected from the samples were compared by calculations of abundance of the ions formed from total ion chromatograms (TIC) using X‐calibur v. 3.1 (Thermo‐Finnigan, San Jose, CA, USA). The identities of volatile compounds were determined by comparing their chromatographic and mass spectral data with those presented in the NIST electronic library, version 2.0 (National Institute of Standard and Technology, USA) and with those of synthetic standards available (for more details see Supporting information, Tables S1 and S2).

### 2.4 Electroantennography (EAG)

Electroantennography was used to determine which volatiles that elicit antennal responses in *A. parviclava* females originating from *G*. *calmariensis*. To achieve clearer electrophysiological responses, we applied direct stimulation using pure compounds, rather than odors from plant‐feeding larvae delivered by gas chromatograph to the antenna. Synthetic standards, analogous to the volatiles emitted from larvae feeding on the plant, were used to stimulate the antenna by puffing them from cartridges into a continuous humidified air stream directed toward the antenna. The synthetic compounds were tested individually as olfactory stimuli on 14 females. Eighteen volatile compounds of three categories were included in the tests, with a control stimulus of 10 μl of cyclohexane: (a) Five compounds that were differentially released by the two larval species, (b) four compounds that were released at equal rates by both species, and (c) nine compounds that were not detected in the headspace collections (see results and Table S2). The latter compounds are common volatiles emitted by plants, which were included as additional controls that were not expected to elicit a response to the antennal measurements. The compounds were tested in a random sequence, and the control stimulus was tested before the first and after the final chemical stimulation, and a mean value was calculated. Test compounds (at a dose of 10 μg) were delivered as samples placed on a 5 × 45 mm strip of filter paper (no. 1, Whatman International Ltd., Maidstone, UK). The solvent (cyclohexane) was allowed to evaporate, and the impregnated filter paper was placed into a 150‐mm long glass Pasteur pipette (Brand Gmbh + Co Kg, Wertheim, Germany) constituting an odor cartridge.

The EAG apparatus (Syntech Ltd., the Netherlands) was linked to a computer (with IDAC‐232 data acquisition interface board) on which the recording, storing, and quantification of EAG responses were performed. The reference electrode, consisting of a glass capillary filled with 0.9% NaCl saline (Ilsanta, Vilnius, Lithuania), was connected to the neck of a detached head from an adult parasitoid. The recording electrode consisted of a similar glass capillary connected to the antennal tip. The stimuli were provided as 0.5 s puffs of air into a continuous humidified air stream at approximately 17 ml/s generated by an air stimulus controller (CS‐55, Syntech, the Netherlands). The peak voltage amplitude (mV) during the puff delivery of each stimulus was recorded as an antennal response (EAG). EAG signals were preamplified (10x) using high‐impedance IDAC‐232 amplifier (Syntech, The Netherlands), recorded, and analyzed using EAG 2000 software (Syntech, the Netherlands). Each stimulation was followed by a minimum of a 1‐min purge period of filtered air to ensure the recovery of antennal receptors.

### 2.5 Statistical analyses

The data from the behavioral assays were analyzed by performing separate generalized linear models with binomial error distribution for each choice combination, with the individual parasitoid as replicate. The volatiles collected from headspace collections (area under the curve) and the responses from the EAG were individually compared between the two species using ANOVAs on ln‐transformed data. When selecting odors from the headspace collection for use in the behavioral assays, we settled on the five compounds with the strongest signal‐to‐noise ratio (as estimated by the F‐ratio and *p*‐values) with the additional condition that the absolute difference in volatile concentration should be more than a 100% increase (for more details see Supporting information, Table S1). This procedure may miss some compounds where the signal‐to‐noise ratio is still large enough to be detected by the parasitoids, as we do not know the signal‐to‐noise ratio needed for detection of odor concentration differences by the antennae. However, the inclusion of too many compounds is less problematic as further testing was performed to examine the actual use of these compounds by the parasitoid. The statistical analyses were performed using R 2.15.2 (R Development Core Team 2012).

## 3 RESULTS

### 3.1 Behavioral assays and identification of volatiles

**Figure 3:**
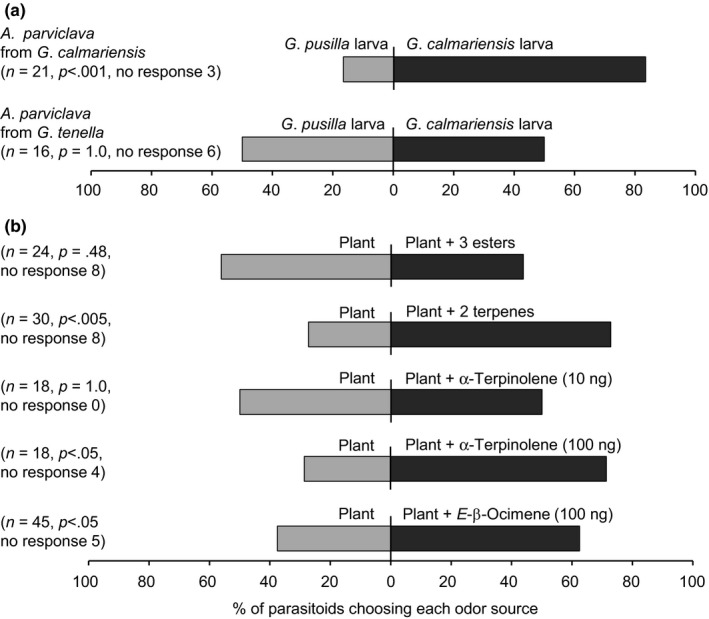
(a) Behavioral responses in *Asecodes parviclava* females originating from *Galerucella calmariensis* and *Galerucella tenella* respectively to *G*. *calmariensis* vs *Galerucella pusilla* larvae feeding on *Lythrum salicaria*. (b) Behavioral responses in *A. parviclava* originating from *G. calmariensis* to host plant (*L*. *salicaria*) odors alone vs a blend of host plant odors and synthetic chemical compounds

**Figure 4:**
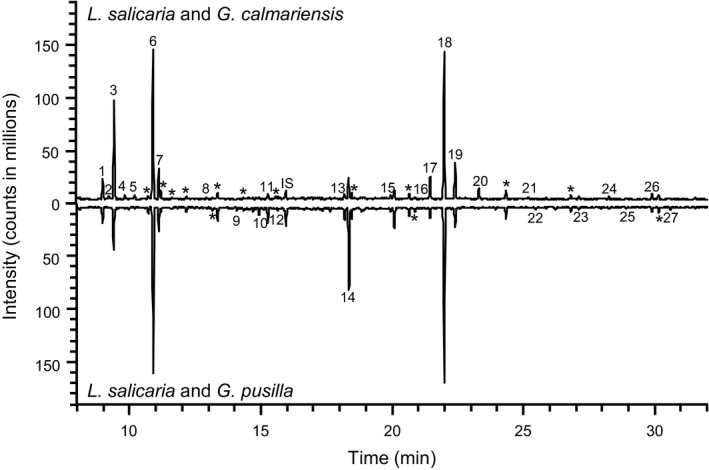
Selected ion chromatogram showing compounds released from *Lythrum salicaria* with feeding larvae of either *Galerucella calmariensis* or *Galerucella pusilla*. Compounds: (1) (*Z*)‐β‐ocimene, (2) γ‐terpinene, (3) (*E*)‐β‐ocimene, (4) *p*‐cymene, (5) α‐terpinolene, (6) (3*E*)‐4,8‐dimethyl‐1,3,7‐nonatriene (DMNT), (7) (*Z*)‐3‐hexenyl acetate, (8) (*Z*)‐3‐hexenol, (9) hexyl 2‐methyl‐butanoate, (10) (*E*)‐2‐hexenyl butanoate, (11) (*Z*)‐3‐hexenyl 3‐methyl‐butanoate, (12) (*E*)‐2‐hexenyl 3‐methyl‐butanoate, (13) β‐elemene, (14) caryophyllene, (15) (*E*)‐β‐farnesene, (16) unidentified sesquiterpene, (17) (*Z*,*E*)‐α‐farnesene, (18) (*E*,*E*)‐α‐farnesene, (19) methyl salicylate, (20) (3*E*,7*E*)‐4,8,12‐trimethyl‐1,3,7,11‐tridecatetraene (TMTT), (21) unidentified sesquiterpene, (22) benzeneethanol, (23) caryophyllene oxide, (24) (*E*)‐nerolidol, (25) hexyl benzoate, (26) (*Z*)‐3‐hexenyl benzoate, (27) eugenol, (IS) internal standard, (*) compound from a blank sample

In the first behavioral assay, *A. parviclava* was given a choice between larvae of *G*. *calmariensis* and *G. pusilla* feeding on the host plant. Our results revealed that parasitoids originating from *G. calmariensis* showed a clear preference for *G*. *calmariensis* larvae (χ^2^ = 17.5, *p* < .001), while parasitoids originating from *G. tenella* did not distinguish between the two larval species (χ^2^ = 3.6, *p* = 1.0; Figure 3a). The analysis of headspace collections of plants and larvae identified five compounds that potentially could be used by parasitoids to differentiate between the volatile profiles of *G. calmariensis* and *G. pusilla* larvae (Figure 4). Host plants in combination with *G. calmariensis* larvae released higher quantities of two terpenes ([*E*]‐β‐ocimene [137% higher] and α‐terpinolene [247% higher]), and host plants in combination with *G. pusilla* larvae released higher quantities of three esters (hexyl 2‐methyl‐butanoate [738% higher], (Z)‐3‐hexenyl 3‐methyl‐butanoate [309% higher] and hexyl benzoate [300% higher]; Figure 4). In the second behavioral assay, we tested the response by *A. parviclava* originating from *G. calmariensis* to these five compounds in mixtures. When first comparing the response to the host plant alone versus the host plant bearing a mixture of the three esters, no significant difference in response could be observed (χ^2^ = 0.5, *p* = .48; Figure 3b). When instead a mixture of the two terpenes was used as treatment, *A. parviclava* preferred plants bearing the synthetic compounds (χ^2^ = 9.4, *p* < .005). When terpenes were tested separately, α‐terpinolene did not elicit a response at 10 ng (χ^2^ = 0, *p* = 1.0), but was attractive when released at a concentration of 100 ng (χ^2^ = 5.3, *p* < .05). A preference was also observed when testing (*E*)‐β‐ocimene at 100 ng (χ^2^ = 5.1, *p* < .05; Figure 3b).

### 3.2 Electroantennography (EAG)

**Figure 5:**
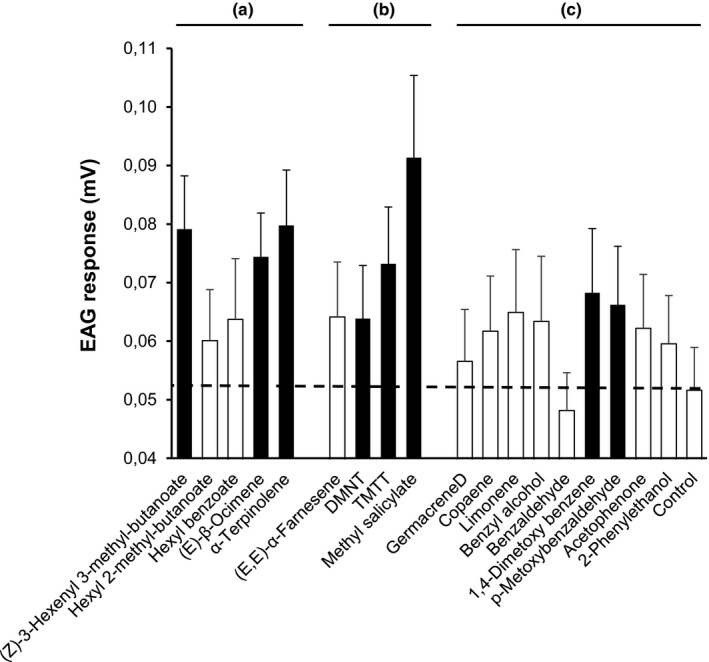
EAG response of *Asecodes parviclava* (mean ± *SE*,* n* = 14) to volatile compounds that were (a) differentially released by the two larval species when feeding on the host plant, (b) released at equal rates by the two larval species, and (c) not detected in the headspace collections (serving as controls). The rightmost bar (and the dotted line) corresponds to the response of control antennae not exposed to any volatile. Black bars indicate responses that significantly differed from the control at *p* < .01

When measuring EAG responses, eight compounds gave a stronger response than the solvent control. The two terpenes ([*E*]‐β‐ocimene, α‐terpinolene) that were differentially released by *G. calmariensis* and *G. pusilla* when feeding on *L. salicaria*, and that were both attractive to *A. parviclava* (Figure 3b), also showed a significant response in the EAG‐analysis (Figure 5). In addition, one ester ([Z]‐3‐hexenyl 3‐methyl‐butanoate) that was differentially released by the two larval species when feeding on the plant, three of four volatiles that were equally released by the two larval species and two of eight control volatiles also elicited responses that were stronger than the odorless control (Figure 5).

## 4 DISCUSSION

It is well known that most parasitoids use volatiles, and particularly host‐induced volatiles, to locate suitable hosts (Vet & Dicke, 1992), but the volatile profiles may not be sufficiently different to allow parasitoids to differentiate among host species using only olfactory cues. The studies suggesting that different herbivore species emit different volatile profiles when feeding on the same plant are restricted to cases where herbivores are distantly related or do not share parasitoids (De Moraes et al., 1998; de Rijk et al., 2013), mainly allowing parasitoids to differentiate between host and nonhost species. On the other hand, parasitoids attacking hosts on different host plant species are documented to use volatile cues for host differentiation (Verschut et al., 2017), but in these cases, the different volatile profiles may be due to differences between the plants themselves. Our study is a rare example showing that parasitoid females use volatile cues to select among hosts feeding on the same host plant and at the same time connect this selection behavior to differences in the volatile profiles. Moreover, our studies involving parasitoid females originating from either *G. calmariensis* or from the alternate host *G. tenella* suggest that the selection behavior of *A. parviclava* may depend on a chemical legacy from earlier life stages.

In this system, the host species vary in resistance to parasitoid attack, and it is notable that *G. calmariensis* (that was selected by parasitoids originating from *G. calmariensis* in the trials) is the species showing the lowest resistance to parasitoid attack (Fors et al., 2014, 2016). In fact, Fors et al. (2014) suggest that most eggs injected in larvae of the other host species used in the trial (*G. pusilla*) are encapsulated and killed. Thus, the capacity to differentiate between the resistant and susceptible host species would enhance offspring survival and increase female fitness (Dupas et al., 2009). While the females in this system certainly have that capacity, the comparison between parasitoids originating from *G. calmariensis* and *G. tenella* suggests that the ability to differentiate between the two hosts may rely on cues experienced during the larval, pupal, or early adulthood stages. This acquired behavior would allow females hatching from the susceptible host to retain the same susceptible host species for their offspring, but without dropping the resistant host from the host repertoire. A similar acquired host selection behavior has been observed in other parasitoid species (van Emden et al., 1996; Stelinski & Liburd, 2005) and may be influential in host race formation (Konig et al., 2015). Host race formation would require that cues inducing host searching behavior for the less accepted host are no longer recognized or avoided (Goldman‐Huertas et al., 2015). However, as the parasitoid females in this system do not have zero fitness when attacking the resistant host, the ability to attack this host could be retained in the population, assuming that it allows survival during times when the susceptible host is not available. Indeed, females of *A. parviclava* lay eggs in *G. pusilla* when no other option is at hand (Fors et al., 2014, 2016). On the other hand, what we are observing may be an evolutionary transition phase, where the ability to differentiate between the two hosts will change from an acquired to a genetically fixed behavior where volatile proportions determine host selection (Davis & Stamps, 2004; De Moraes et al., 1998).

In the current system, an alternative evolutionary pathway would be that parasitoid females evolve a higher virulence, allowing them to attack the resistant host successfully. Studies on the population variation in the interactions between *A. parviclava* and its host species also suggest that there is population variation in parasitoid virulence. Parasitoids originating from sites with a relatively high proportion of *G. pusilla* (the resistant host) have a higher virulence (Fors et al., 2016). It is possible that there is also genetic differentiation in the ability of females to select among the host species, but we currently have no data to support such a pattern. Similar variations in virulence and host recognition traits are known from *Drosophila* and their parasitoids (Dubuffet et al., 2009; Dupas et al., 2009), as well as from some other systems (Catherine, Schulthess, & Stephane, 2010).

Previous work on host–parasitoid interactions provides little guidance on the relative role of virulence and preference traits in parasitoid evolution. For example, most theoretical studies on evolution in host–parasitoid systems have focused on immunity and virulence traits in monophagous parasitoid species (Lapchin, 2002; Sasaki & Godfray, 1999; Sisterson & Averill, 2004), where parasitoid selection among hosts is a less relevant trait. Empirical studies, on the other hand, have more commonly examined the evolution of immunity and virulence in systems with oligophagous parasitoids. One example that included preference is the study by Dubuffet et al., 2006; showing not only that preference of *Leptopilina boulardi* between *Drosophila melanogaster* and *Drosophila yakuba* match differences in host virulence, but also that this matching is differentiated among populations depending on the dominance of the two *Drosophila* species (Dubuffet et al., 2006). In Mediterranean areas, where *D. melanogaster* is the most common species, *L. boulardi* has the stronger virulence against this species and females select *D. melanogaster* for egg laying. In central African populations, on the other hand, *D. yakuba* is the most abundant species. *L. boulardi* in these areas then has the stronger virulence against this species and females select *D. yakuba* for egg laying. Differences in host selection behavior due to different geographical origin have been observed also in other parasitoid species, for example, the *Drosophila* parasitoids *Leptopilina clavipes* and *Asobara tabida* (Kraaijeveld, Nowee, & Najem, 1995; Pannebakker, Garrido, Zwaan, & van Alphen, 2008). In these species, parasitoid females with a higher ability to prevent encapsulation more willingly accepted a better‐defended host, when given a choice between larvae of two host species.

In the current system, the olfactory cues that are the most likely candidates for the observed parasitoid preference were two terpenoids, which were produced in the highest amounts by the preferred host, *G. calmariensis*. In contrast, the three esters that were produced in the highest amounts by the less selected host, *G. pusilla*, did not seem to affect parasitoid selection either positively or negatively, and only one of the esters ([Z]‐3‐Hexenyl 3‐methyl‐butanoate) caused a response in the electroantennogram (Figures 4, 5, Table S1 and S2). To definitely identify the role of the terpenoids in the host selection behavior by *A. parviclava*, additional studies would be needed that compare responses in a gradient of signal‐to‐noise ratios. In addition to these compounds, the electroantennogram identified three additional compounds (methyl salicylate, DMNT, and TMTT) that are emitted in equal amounts by both larvae–plant combinations and which are detected by *A. parviclava* antennae. At least two of these compounds (DMNT and TMTT) show very small concentration differences between the two larval species and are less likely candidates for explaining host selection of *A. parviclava*. Methyl salicylate is perhaps more interesting as the volatile profile of *G. calmariensis* contains a 74% higher concentration of this compound. However, as it was still under the threshold (less than a 100% increase), this compound was not included in the Y‐tube experiments. In any case, even though the role of these compounds in the host finding and selection behavior by *A. parviclava* is unknown, the fact that they are detected by the insect antennae may warrant further studies.

We may also note that the compounds detected by *A. parviclava* are mainly those that are emitted at higher concentrations by *G. calmariensis*, potentially with the exception of the ester (Z)‐3‐Hexenyl 3‐methyl‐butanoate. We may of course have missed some important compound, but this observation raises the question of what a parasitoid originating from *G. pusilla* would use as a chemical legacy. Would these parasitoids use the same cues, but then as a repellent instead of an attractant, or will they base the selection on another cue that is emitted in higher concentrations by feeding *G. pusilla* larvae? We are not aware of any study that has examined differences in cues used by parasitoid females when originating from different host species.

The cause for the different volatile profiles emitted by *G. calmariensis* and *G. pusilla* is yet unclear. The most likely hypothesis is that the different volatile profiles arise due to differences in induced volatiles from feeding damage by the two larval species. However, we cannot exclude the possibility that the differences in volatile profiles arise due to differences in larval odors. The latter hypothesis is less likely, as the amounts of compounds released by damaged plants are usually far higher than the amounts released directly by larvae. If that interpretation is correct, different volatile profiles may arise either because of differences in damage caused by the feeding larvae or because of differences in the chemical composition of saliva from the two larval species, which then interact differently with the plant chemistry.

In conclusion, our work is important for understanding host selection in the *Galerucella‐Asecodes* system and the evolutionary pathways underlying host race formation and speciation in this system. We also believe that further studies of these processes in this system may provide general guidance on the interactive role of host selection behavior and evolution of parasitoid virulence in the host–parasitoid coevolutionary process. The fact that we have previously found population differentiation in at least one trait, that is, parasitoid virulence, suggests an ongoing evolutionary process. However, in comparison with the *Drosophila*–parasitoid system, the population variation in the *Galerucella‐Asecodes* system occurs on much smaller spatial scales. Moreover, in contrast to the *Drosophila* system (Dubuffet et al., 2006), we have not yet identified any costs involved in evolving increased parasitoid virulence. Thus, it is still possible that the population differentiation is maintained by population isolation and that the virulence traits would sweep through a population following population mixing. In this context, it may not be surprising that the host selection behavior does not get fixed in the population but rather depends on learning during earlier life stages.

## CONFLICT OF INTEREST

None declared.

## AUTHORS’ CONTRIBUTIONS

PAH, LF, TAV, and RM conceived and designed the experiments. LF, TAV, LBC, and RM performed the experiments. LF, PAH, and RM analyzed the data. LF and PAH wrote the manuscript; other authors provided editorial advice.

## DATA ACCESSIBILITY

Data files supporting this study are available in the Dryad Digital Repository: https://doi.org/10.5061/dryad.qj812.
