# Supplementary material for: Selection by parasitoid females among closely related hosts based on volatiles: Identifying relevant chemical cues
Source: Ecol Evol. 2018 Feb 19;8(6):3219–28. doi: 10.1002/ece3.3877 (PMC5869356; doi:10.1002/ece3.3877)
Supplement: Supplementary file 1 [file ECE3-8-3219-s001.docx]

**Selection by parasitoid females among closely related hosts based on volatiles: Identifying relevant chemical cues**

Lisa Fors^1,3^, Raimondas Mozuraitis^1,2,3^, Laima Blažytė-Čereškienė^2^, Thomas A. Verschut^1^ and Peter A. Hambäck^1^

^1^Department of Ecology, Environment and Plant Sciences, Stockholm University, SE-106 91, Sweden

^2^Laboratory of Chemical and Behavioural Ecology, Institute of Ecology, Nature Research Centre, Akademijos st. 2, LT-08412 Vilnius, Lithuania

^3^Current address: Department of Zoology, Stockholm University, SE-106 91, Sweden

Corresponding author: Lisa Fors

Stockholm University, SE-106 91 Stockholm, Sweden

[lisa.fors@su.se](mailto:lisa.fors@su.se)

00468-164279

**Supporting information:**

**Table S1**

**Table S2**

**Table S1.** Identified volatile compounds from headspace of *Lythrum salicaria* plants infested with larvae of either *Galerucella calmariensis* or *G. pusilla*.

|  |  |  |  | | | |  | | |  |  | Intensity (area under the peak in millions) | | | |
| --- | --- | --- | --- | --- | --- | --- | --- | --- | --- | --- | --- | --- | --- | --- | --- |
| No | Compound name | | | CAS No | Identity | RI | | *G. calmariensis* + plant | | | | | *G. pusilla* + plant | |  |
| 1 | (*Z*)-β-Ocimene | | | 3338-55-4 | MT | 1235 | | | 113.9 (SE+ = 29.7, SE- = 23.5) | | | | | 73.2 (SE+ = 19.1, SE- = 15.1) | |
| 2 | γ-Terpinene | | |  | MT | 1245 | | | 3.8 (SE+ = 1.8, SE- = 1.2) | | | | | 1.0 (SE+ = 0.5, SE- = 0.3) | |
| 3 | **(*E*)-β-Ocimene** | | | 3779-61-1 | MT | 1253 | | | 247.8 (SE+ = 105.2, SE- = 73.8) | | | | | 104.4 (SE+ = 29.2, SE- = 22.8) | |
| 4 | p-Cymene | | | 99-87-6 | AR | 1268 | | | 5.1 (SE+ = 3, SE- = 1.9) | | | | | 4.7 (SE+ = 2.7, SE- = 1.7) | |
| 5 | ** α-Terpinolene** | | | 586-62-9 | MT | 1282 | | | 12.5 (SE+ = 2.8, SE- = 2.3) | | | | | 3.6 (SE+ = 0.9, SE- = 0.7) | |
| 6 | DMNT | | | 19945-61-0 | HT | 1307 | | | 226.6 (SE+ = 62.5, SE- = 49) | | | | | 304.6 (SE+ = 82.8, SE- = 65.1) | |
| 7 | (*Z*)-3-Hexenyl acetate | | | 3681-71-8 | ES | 1316 | | | 78.4 (SE+ = 24.9, SE- = 18.9) | | | | | 108.3 (SE+ = 51.9, SE- = 35.1) | |
| 8 | (*Z*)-3-Hexenol | | | 928-96-1 | AL | 1385 | | | 4.8 (SE+ = 1.1, SE- = 0.9) | | | | | 4.6 (SE+ = 1.3, SE- = 1) | |
| 9 | **Hexyl 2-methyl-butanoate** | | | 10032-15-2 | ES | 1428 | | | 0.8 (SE+ = 0.5, SE- = 0.3) | | | | | 6.7 (SE+ = 5.2, SE- = 2.9) | |
| 10 | (*E*)-2-Hexenyl butanoate | | | 53398-83-7 | ES | 1462 | | | 9.6 (SE+ = 6.7, SE- = 4) | | | | | 21.5 (SE+ = 16.9, SE- = 9.5) | |
| 11 | **(*Z*)-3-Hexenyl 3-methyl-butanoate** | | | 35154-45-1 | ES | 1475 | | | 19.8 (SE+ = 10.7, SE- = 6.9) | | | | | 80.9 (SE+ = 57.4, SE- = 33.6) | |
| 12 | (*E*)-2-Hexenyl 3-methyl-butanoate | | | 68698-59-9 | ES | 1489 | | | 4.3 (SE+ = 3, SE- = 1.7) | | | | | 4.7 (SE+ = 3.8, SE- = 2.1) | |
| 13 | β-Elemene | | | 515-13-9 | ST | 1589 | | | 3.5 (SE+ = 3.3, SE- = 1.7) | | | | | 1.8 (SE+ = 1.7, SE- = 0.9) | |
| 14 | Caryophyllene | | | 87-44-5 | ST | 1596 | | | 124.9 (SE+ = 47, SE- = 34.1) | | | | | 103.8 (SE+ = 168.6, SE- = 64.2) | |
| 15 | (*E*)-β-Farnesene | | | 18794-84-8 | ST | 1667 | | | 5.7 (SE+ = 5.8, SE- = 2.9) | | | | | 22.1 (SE+ = 16.6, SE- = 9.5) | |
| 16 | unidentified sesquiterpene | | |  | ST | 1719 | | | 2.8 (SE+ = 5.7, SE- = 1.9) | | | | | 1.1 (SE+ = 2.3, SE- = 0.8) | |
| 17 | (*Z*,*E*)-α-Farnesene | | | 26560-14-5 | ST | 1727 | | | 21.5 (SE+ = 6.4, SE- = 5) | | | | | 14.2 (SE+ = 5.6, SE- = 4) | |
| 18 | (*E*,*E*)-α-Farnesene | | | 502-61-4 | ST | 1752 | | | 222.8 (SE+ = 68.5, SE- = 52.4) | | | | | 305.0 (SE+ = 112, SE- = 81.9) | |
| 19 | Methyl salicylate | | | 119-36-8 | AR | 1769 | | | 80.4 (SE+ = 15.7, SE- = 13.1) | | | | | 46.1 (SE+ = 15.3, SE- = 11.5) | |
| 20 | TMTT | | | 62235-06-7 | HT | 1808 | | | 5.0 (SE+ = 3.3, SE- = 2) | | | | | 4.6 (SE+ = 3.1, SE- = 1.8) | |
| 21 | unidentified sesquiterpene | | |  | ST | 1891 | | | 1.6 (SE+ = 0.8, SE- = 0.5) | | | | | 2.1 (SE+ = 0.9, SE- = 0.7) | |
| 22 | Benzeneethanol | | | 60-12-8 | AR | 1903 | | | 9.4 (SE+ = 5.2, SE- = 3.4) | | | | | 6.5 (SE+ = 4, SE- = 2.5) | |
| 23 | Caryophyllene oxide | | | 1139-30-6 | OST | 1982 | | | 6.7 (SE+ = 1.4, SE- = 1.2) | | | | | 7.9 (SE+ = 2.4, SE- = 1.9) | |
| 24 | (*E*)-Nerolidol | | | 40716-66-3 | OST | 2037 | | | 4.3 (SE+ = 1.1, SE- = 0.9) | | | | | 4.2 (SE+ = 1, SE- = 0.8) | |
| 25 | **Hexyl benzoate** | | | 6789-88-4 | ES | 2072 | | | 0.5 (SE+ = 0, SE- = 0) | | | | | 2.0 (SE+ = 1.1, SE- = 0.7) | |
| 26 | (*Z*)-3-Hexenyl benzoate | | | 25152-85-6 | ES | 2120 | | | 12.8 (SE+ = 13.1, SE- = 6.5) | | | | | 23.5 (SE+ = 27.1, SE- = 12.6) | |
| 27 | Eugenol | | | 97-53-0 | AR | 2158 | | | 3.5 (SE+ = 2.1, SE- = 1.3) | | | | | 6.4 (SE+ = 4.2, SE- = 2.5) | |

Bold compounds indicate those that were collected in different amounts from the two sample types. Values and standard errors are back-transformed from log-transformed mean values. DMNT (3*E*)-4,8-Dimethyl-1,3,7-nonatriene; TMTT (3*E*,7*E*)-4,8,12-Trimethyl-1,3,7,11-tridecatetraene; CAS No Chemical Abstract Service number; RI retention index, MT monoterpene; AR aromatic compound; HT homoterpene; ES ester; AL alcohol; ST sesquiterpene; OST oxygenated sesquiterpene

**Table S2.** Purity and source of synthetic compounds used in EAG and behavioural experiments

| Compound | CAS No | Identity | Purity (%) | Source |
| --- | --- | --- | --- | --- |
| (*Z*)-3-Hexenyl 3-methyl-butanoate | 35154-45-1 | ES | 97 | Sigma-Aldrich |
| Hexyl 2-methyl-butanoate | 10032-15-2 | ES | 97 | Sigma-Aldrich |
| Hexyl benzoate | 6789-88-4 | ES | 98 | Sigma-Aldrich |
| (*E*)-β-Ocimene | 3779-61-1 | MT | 97 | BOC Science |
| α-Terpinolene | 586-62-9 | MT | 98 | TCI Europe Research Chemicals |
| (*E*,*E*)-α-Farnesene | 502-61-4 | ST | 98 | BOC Science |
| DMNT | 19945-61-0 | ST | 98 | KTH |
| TMNT | 62235-06-7 | HT | 98 | KTH |
| Methyl salicylate | 119-36-8 | AR | 98 | Alfa Aesar |
| Germacrene D | 23986-74-5 | ST | 99 | KTH |
| Copaene | 3856-25-5 | ST | 97 | BOC Science |
| Limonene | 138-86-3 | MT | 97 | TCI Europe Research Chemicals |
| Benzyl alcohol | 100-51-6 | AR | 99 | Alfa Aesar |
| Benzaldehyde | 100-52-7 | AR | 99 | Sigma-Aldrich |
| 1,4-Dimetoxy benzene | 150-78-8 | AR | 99 | Sigma-Aldrich |
| *p*-Metoxybenzaldehyde | 123-11-5 | AR | 98 | Sigma-Aldrich |
| Acetophenone | 98-86-2 | AR | 99 | Sigma-Aldrich |
| 2-Phenylethanol | 98-85-1 | AR | 98 | Sigma-Aldrich |

DMNT (3*E*)-4,8-Dimethyl-1,3,7-nonatriene; TMTT (3*E*,7*E*)-4,8,12-Trimethyl-1,3,7,11-tridecatetraene; CAS No Chemical Abstract Service number; ES ester; MT monoterpene; ST sesquiterpene; HT homoterpene; AR aromatic compound; KTH compounds were synthesized or isolated from a natural source and afterwards purified at the Department of Chemistry, Royal Institute of Technology, Stockholm.
